# Anthelmintic and Hepatoprotective Activities of the Green-Synthesized Zinc Oxide Nanoparticles Against *Parascaris equorum* Infection in Rats

**DOI:** 10.1007/s11686-023-00728-4

**Published:** 2023-12-06

**Authors:** Sara Bayoumi Ali, Ayman Saber Mohamed, Sohair R. Fahmy, Manal El–Garhy, Mohamed R. Mousa, Fathy Abdel-Ghaffar

**Affiliations:** 1 Zoology Department, Faculty of Science, Cairo University, Giza, Egypt; 2 Pathology Department, Faculty of Veterinary Medicine, Cairo University, Giza, Egypt

**Keywords:** *Parascaris equorum*, Zinc oxide nanoparticles, Oxidative stress, Green synthesis

## Abstract

**Main conclusions:**

Green-synthesized zinc oxide nanoparticle is a promising treatment modality against parasitic infection through its powerful anthelmintic, antioxidant, healing promotion, and anti-inflammation effects.

**Background:**

Nanoparticles have many properties, depending on their size, shape, and morphology, allowing them to interact with microorganisms, plants, and animals.

**Objectives:**

Investigation of the therapeutic effects of green-synthesized zinc oxide nanoparticles (ZnO NPs) on *Parascaris equorum* infection in rats.

**Methods:**

Thirty-six rats were divided into two divisions: the first division is noninfected groups were allocated into three groups. Group 1: Control, group 2: ZnO NPs (30 mg/kg), and group 3: ZnO NPs (60 mg/kg). The second division is infected groups were allocated into three groups. Group 1: vehicle, group 2: ZnO NPs (30 mg/kg), and group 3: ZnO NPs (60 mg/kg).

**Findings:**

Ten days post-infection, two larvae per gram of liver tissue were present in the vehicle group compared to the control group. No larvae were recovered from ZnO NPs (30 mg/kg), and one larva/g.tissue from ZnO NPs (60 mg/kg)-treated groups compared to untreated infected animals. Green-synthesized ZnO NPs caused a significant decrease in liver functions, low-density lipoprotein (LDL), cholesterol, triglycerides, malondialdehyde (MDA), and nitric oxide (NO). While it caused a significant increase in hemoglobin (HB), high-density lipoprotein (HDL), butyrylcholinesterase (BCHE), glutathione (GSH), catalase (CAT), and glutathione S-transferase (GST) in infected treated rats. The histological inflammation and fibroplasia scores showed a significant enhancement during the treatment with ZnO NPs (30, 60 mg/kg) compared to the infected untreated animals that scored the highest pathological destruction score. Immunohistochemical markers of NF-κB showed a significant decrease during the treatment with ZnO NPs (30, 60 mg/kg) compared to the infected untreated animals.

## Introduction

Equine ascariasis is an endoparasitic disease that constitutes a public health problem worldwide, caused by the larvae of *Parascaris equorum*, which are frequently present in the small intestine of the horse, ass, mule, zebra, and other equids. In severe cases, the infection with *P. equorum* is associated with unthriftiness, respiratory signs, or intestinal impaction. In some cases, ascarids can lead to an obstruction and rupture of the small intestine or the death of foals [1, 2]. Infection can be diagnosed by detecting eggs in feces, but this is only possible after the worms are fully mature [3]. Foals less than six months old are most susceptible to the infection and the most fabulous egg production source. The female of *P. equorum* lays up to 200,000 eggs per day and these eggs are passed to the outside in the manure [4]. The infective stage is a larvated egg (containing a second stage larva [L3]); development requires approximately 10–14 days at temperatures of 25–35 °C [5]. Larvated eggs survive in the environment for up to five or ten years, and infection is acquired through accidental ingestion of eggs. Larvae emerge from eggs within the alimentary tract of a horse, migrate through the liver and lungs, and may attack other tissues for 2–4 weeks, and then return to the small intestine fourth stage (L4). Ascarids mature progressively in the small intestine and achieve patency about 75–80 days after infection [6]. Visceral larva migrans (VLM), caused by nematode larvae, pass through different organs such as the liver, lung, kidney, and spleen. The duration and severity of liver involvement were greater in comparison with other organs and gradually extended to the lungs, heart, kidney, brain, and muscles. This suggests the migratory nature of larvae from the intestine to other organs which commenced through the liver [7]. The liver plays a critical role in maintaining homeostasis through metabolism, synthesis of proteins, formation of bile, clotting factors, and detoxification of various compounds. It is an immunotolerant organ, that is an ideal place for parasites to hide from the immune system [8]. Numerous helminths (cestodes, trematodes, and nematodes) parasitize the liver [5]. Because of its function as a filter of the portal venous blood flow from the gut, it is the primary site for tissue-dwelling helminths transmitted orally [8]. Improvement in biomedical nanotechnology leads to many exciting applications such as drug discovery, drug delivery, and gene/protein delivery [9]. Nanoparticles (NPs) have different properties, depending on their size, shape, and morphology, allowing them to interact with microorganisms, plants, and animals [10]. Moreover, nanoparticles are more highly absorbed into the respiratory, skin, and gastrointestinal systems than micron-sized particles [11]. Recently, metal nanoparticles (NPs) and their oxides have many applications in the medical and industrial fields [12]. Zinc (Zn) is a fundamental element for human and animal health but it is toxic to microorganisms. ZnO-based nanomaterials have developed enormous importance in nanomedicine and have practical biomedical applications due to their low cost, high stability, wide bandgap, small size, and physicochemical properties [10]. The main drawbacks of physical and chemical methods for synthesizing nanomaterials are using toxic compounds, consuming a high amount of energy, and generating hazardous wastes. So, the green synthesis of nanoparticles is considered as an eco-friendly technology because it does not involve poisonous compounds and is safe in handling [13]. In the current study, green-synthesized zinc oxide nanoparticles were synthesized from egg white. Egg white is valuable to the human body with high nutritional quality, as well as it has efficient properties, such as its solubility in water and tendency to associate with metal ions in solution [14]. Metal ions such as Mn^2+^/Mn^3+^, Fe^2+^/Fe^3+^, Cu^2+^, Zn^2+^, and Ni^2+^ have been combined with egg white to obtain new nanoparticles. Egg white stabilizes the small nanoparticles and reduce the surface relaxation of the forming nanoparticles resulting in smaller values of the lattice parameters [15].

Zinc is an essential trace element in humans and relatively nontoxic. It plays an important role in biological processes such as growth and division of cells, osteogenesis, and immune response [16]. It appears that zinc has different ways to protect liver against damage and fibrosis. Zinc can reduce transforming growth factor beta-induced epithelial differentiation and fibroblast activation, which are common features of tissue fibrosis [17]. As ZnO NPs possess various therapeutic properties, they have potent hepatoprotective activity against liver damage. ZnO NPs can protect cell membrane integrity against oxidative stress damage and lowering the biochemical parameters (ALT, AST, and GGT) and expression of inflammatory markers (TNF-α and IL-6) to normal level, thereby improving the liver pathology [18]. In addition, ZnO NPs could inhibit rat liver fibrosis development as appeared from a significant decrease of hepatic hydroxyproline level and decrease of α-SMA-positive cells and collagen bundles [19]. Thereby, the present study was designed to use the rats as a novel model for *P. equorum* infection and evaluate the impact of *P. equorum* larvae on liver physiology and histopathology; moreover, investigating the therapeutic effect of green-synthesized zinc oxide nanoparticles against equine ascariasis in rats.

## Materials and Methods

### Synthesis of Green-Synthesized ZnO NPs

The synthesis of green-synthesized ZnO NPs using egg albumin as a biotemplate (denoted as “ZnO NPs”) was performed according to the previously reported method [20]. In brief, freshly extracted 30 mL egg albumin (5 mg/mL) was mixed dropwise into 70 mL aqueous 0.25M zinc acetate [Zn (CH_3_COOH)_2_.2H2O] solution. The mixture was stirred 20 min at room temperature and precipitated by the addition of the ammonia (NH3) at ~ pH 7.0 and centrifuged at 5000 rpm for 10 min. The obtained pellet was washed twice carefully with sterile distilled water and dried in the vacuum oven. The obtained dried powder of ZnO NPs was subjected to sintering at 400 °C for 3 h, collected in dry tubes, and stored in refrigerator at 4 °C until used.

### Purification of Green-Synthesized ZnO NPs

The resulting ZnO NPs were purified in a mixture of methanol, hexane, and isopropanol with a volume ratio equal to 1:5:1. ZnO NPs were added to methanol-producing ZnO methanol colloids. White ZnO nanoparticles precipitated immediately after adding hexane and isopropanol into the ZnO methanol colloids. The mixture was kept at 0 °C overnight until ZnO NPs were entirely precipitated and settled down to the bottom. After centrifugation and removing the supernatant, the precipitated ZnO was redispersed in methanol by handshaking. The above operations were repeated several times to wash the ZnO NPs in methanol [21].

### Characterization of Green-Synthesized ZnO NPs

#### Ultra-Violet–Visible Spectroscopy

The optical property of green-synthesized ZnO nanoparticles were observed from the absorption spectra of nanoparticles synthesized at various temperatures and concentrations. UV–Vis spectra were measured using Varian; Cary 5000 UV–visible spectrophotometer with a wavelength in the range of 200–800 nm at room temperature [22].

#### X-Ray Diffraction Analysis

Crystallographic properties of ZnO NPs were explored using a PANalyticalX’Pert X-ray diffractometer equipped with a nickel filter using copper (Cu) Ka radiation as an X-ray source. The size of the particles was calculated using Scherrer’s formula as follows: *d* = *Kλ*/*β* cos*θ*, where *d* is the crystalline size, *K* = 0.89 is the shape factor, *k* is the X-ray wavelength of Cu Ka radiation (0.154 nm), *θ* is the Bragg diffraction angle, and *β* is the full width at half maximum of the respective diffraction peak [23].

#### Transmission Electron Microscopy (TEM)

The morphological study of the green-synthesized zinc oxide nanoparticles was performed by TEM, which was achieved at an accelerated voltage of 120 kV (JEM- JEM 2100F; JEOL Ltd, Tokyo, Japan) (Electron Microscopy Unit, Faculty of Agriculture). ZnO NPs were dissolved in distilled water and then sonicated by a probe sonicator until a colloidal solution is formed. After that, a small drop of colloidal suspension (usually about 5 µL) is put on the TEM grid and let it dry at room temperature. The grid can then be directly observed in a TEM once the medium has evaporated. To get the best result, avoid high concentrations [22].

### Collection of Worms

Freshly isolated gastrointestinal tracts of young domestic donkeys (< 5 years), *Equus ferus caballus* Linnaeus 1758 (Family Equidae) necropsied at the Faculty of Veterinary Medicine were collected and helminthological examined specifically for the collection of equine nematodes. The various organs are separated and placed individually in shallow plastic jars. The gastrointestinal tract contents are put into separate theological plastic containers and examined for helminth parasites standard methods [24]. After dissection and isolation of the gastrointestinal tracts in the slaughterhouse, adult worms were collected and transported in a flask containing Goodwin’s solution to the laboratory.

### * In vitro P. equorum* Eggs Embryonation

Adult *P. equorum* worms were recovered from the intestines of naturally infected donkeys. *P. equorum* females were dissected in Petri dishes containing acidified water (pH = 3), and the uteri were removed and cut open to release the eggs. The recovered eggs were centrifugated at 1500 rpm for 5 min. The pellet containing the eggs was transferred to a glass beaker containing approximately 200 mL of 0.5% formalin covered with a hydrophobic cotton plug. The beaker was placed in an incubator at 28 °C and manually agitated twice daily to ensure the oxygenation of eggs to promote larvae development up to the third stage. After 20 days (the length of time required for third-stage larval formation), the embryonated eggs were washed three times in 0.9% saline solution to remove the formalin solution and prepared for infection in the rats [25]. Infective *P. equorum* eggs were collected and kept at 4 °C until use. On the day of infection, a viability test was performed. Only well-developed, motile, and unhatched larvated eggs were observed by microscope and counted as viable and infective [26, 27].

### Ethical Approval and Experimental Animals

Experimental protocols used in this experiment were approved by Institutional Animal Care and Use Committee (IACUC) at Faculty of Science, Cairo University, Egypt (CUI/F/85/20). All the experimental procedures were carried out following international guidelines for the care and use of laboratory animals. Adult male Wistar rats (*Rattus norvegicus*) with an average body weight of 150–160 ± 5 g was bought, grouped, and housed in polyacrylic cages in well-ventilated animal house. Rats were maintained in a friendly environment of 12-h/12-h light–dark cycle at room temperature (22–25 °C). They were allowed to adapt to the environment for 7 days before the experiment and fed standard chow pellets and water ad libitum.

### Acute Toxicity Study LD_50_

Ten male rats were used for the determination of the LD_50_. The rats were divided into five groups (A–E) of two animals each. Group A: Control animals were administrated orally distilled water. Groups (B–E) were given orally different doses of ZnO NPs (10, 100, 300, and 600 mg/kg), respectively. The rats were observed for one-hour post-administration and then 10 min every two hours interval for 24 h. The animal was monitored for any difference in behaviors such as paw licking, fatigue, semi-solid stool, salivation, writhing, and loss of appetite, in addition to mortality. LD_50_ was calculated from the following formula: LD_50_ = (*M*_0_ + *M*_1_)/2, where *M*_0_ is the highest dose of green-synthesized ZnO NPs that caused no mortality; *M*_1_ is the lowest dose of green-synthesized ZnO NPs that caused mortality [28].

### Experimental Design

Animals (36 rats) were assigned into two main divisions as follows: **The first division** (18 rats) was allocated into three groups. Group 1: Control, 6 rats received a single oral dose of saline and then received distilled water for 10 days. Group 2: ZnO NPs (30 mg/kg), 6 rats received orally 1/20 LD_50_ ZnO NPs for 10 consecutive days. Group 3: ZnO NPs (60 mg/kg), 6 rats received orally 1/10 LD_50_ ZnO NPs for 10 consecutive days. **The second division** (*P. equorum* infection division, 18 rats) was allocated into three groups. Group 1: Vehicle, 6 rats received a single oral dose of 1000 *P. equorum* eggs [25] and then received distilled water for 10 days. Group 2: ZnO NPs (30 mg/kg), 6 rats received a single oral dose of 1000 *P. equorum* eggs and were then administered orally 1/20 LD_50_ of ZnO NPs for 10 consecutive days. Group 3: ZnO NPs (60 mg/kg), 6 rats received a single oral dose of 1000 *P. equorum* eggs and then administered orally 1/10 LD_50_ of ZnO NPs for 10 consecutive days.

### Animal Handling

At the end of the experiment, the animals were anesthetized by intraperitoneal injection of sodium pentobarbital (50 mg/kg body weight) [29]. Blood samples were collected by exsanguination then they were centrifuged at 3000 rpm for 20 min. The clear non-hemolyzed sera were aspirated into sterilized Eppendorf tubes for each rat. The sera were kept in a deep freezer at – 80 °C, pending biochemical analysis. After blood collection, the rats were dissected, and the liver was quickly removed and washed with physiological saline to remove traces of blood and divided into three pieces, 1st part was homogenized for the biochemical analyses, 2nd part was immersed in 10% formalin solution for histopathological examination, and 3rd part was examined for *P. equorum* larvae recovery.

### Larvae Recovery and Counting

The acid-isolating technique was used to recover *P. equorum* larvae from the hepatic tissues; the infected livers were weighted and added in a 5 mL test tube containing 2 mL of 0.5% HCL. Test tubes were placed in an incubator at 37 °C for 24 h to allow for the precipitation of the larvae. After the incubation period, infected tissues were removed from test tubes. HCL-containing larvae were centrifugated for 5 min at low speed. The supernatant was removed, and the pellet was examined under the light microscope [25].

### Biochemical Markers

The collected sera were used for determining the serum total cholesterol (TC), serum triglycerides (TG), serum high-density lipoprotein (HDL), serum low-density lipoprotein (LDL), aspartate aminotransferase (AST), alanine aminotransferase (ALT), alkaline phosphatase (ALP), gamma-glutamyltransferase (GGT), total protein, albumin, bilirubin, butyrylcholinesterase (BCHE), hemoglobin (Hb), and bilirubin according to the manufacturer’s instructions.

### Organ Homogenate Preparation and Oxidative Stress Markers

Liver tissues were weighted and homogenized (10% w/v) in ice-cold 0.1 M Tris–HCl buffers (pH 7.4). The homogenate was centrifuged at 3000 rpm for 10 min at 4 °C and the supernatant was used for biochemical analyses. The supernatant of the homogenate of the liver was used for biochemical analysis according to the manufacturer’s instructions. Malondialdehyde (MDA), reduced glutathione (GSH), glutathione-s-transferase (GST), nitric oxide (NO), and catalase (CAT) were determined.

### Histopathology

Liver tissue specimens were kept in neutral buffered formalin (10%) followed by processing in alcohol and xylene. Melted paraffin wax was used for embedding and 5 µm sections were cut and stained with hematoxylin and eosin stain (H&E) for light microscopy and picrosirius red stain (PSR) for normal bright field and polarized light examination to evaluate the degree of fibroplasia that was quantified as area percent [30].

### Immunohistochemistry

Liver tissue sections were cut on adhesive glass slides for immune staining. Briefly, tissue slides were subjected to epitope retrieval step followed by peroxidase blocking. Slides were incubated with primary anti-NF-κβ (at a dilution of 1:100 for 2 h at room temperature) followed by washing then a Mouse/Rabbit Immuno-Detector DAB HRP kit (Bio SB, CA, USA) was used as manufacturer instructions to develop and visualize the reaction. Negative control slides were obtained by escaping the primary antibody step. Positive expression was quantified as area percent using Cell Sens dimensions Olympus software (Olympus, Tokyo, Japan) [31].

### Morphological Identification of *P. equorum* Larvae by Scanning Electron Microscopy (SEM)

Recovered larvae from hepatic tissues were fixed in 3% glutaraldehyde (pH 7.4) buffered in 0.1 M sodium cacodylate buffer for SEM as described by dos Santos *et al*. [25]

## Results

### Characterization of ZnO NPs

#### Ultra-Violet–Visible Spectroscopy

**Fig. 1:**
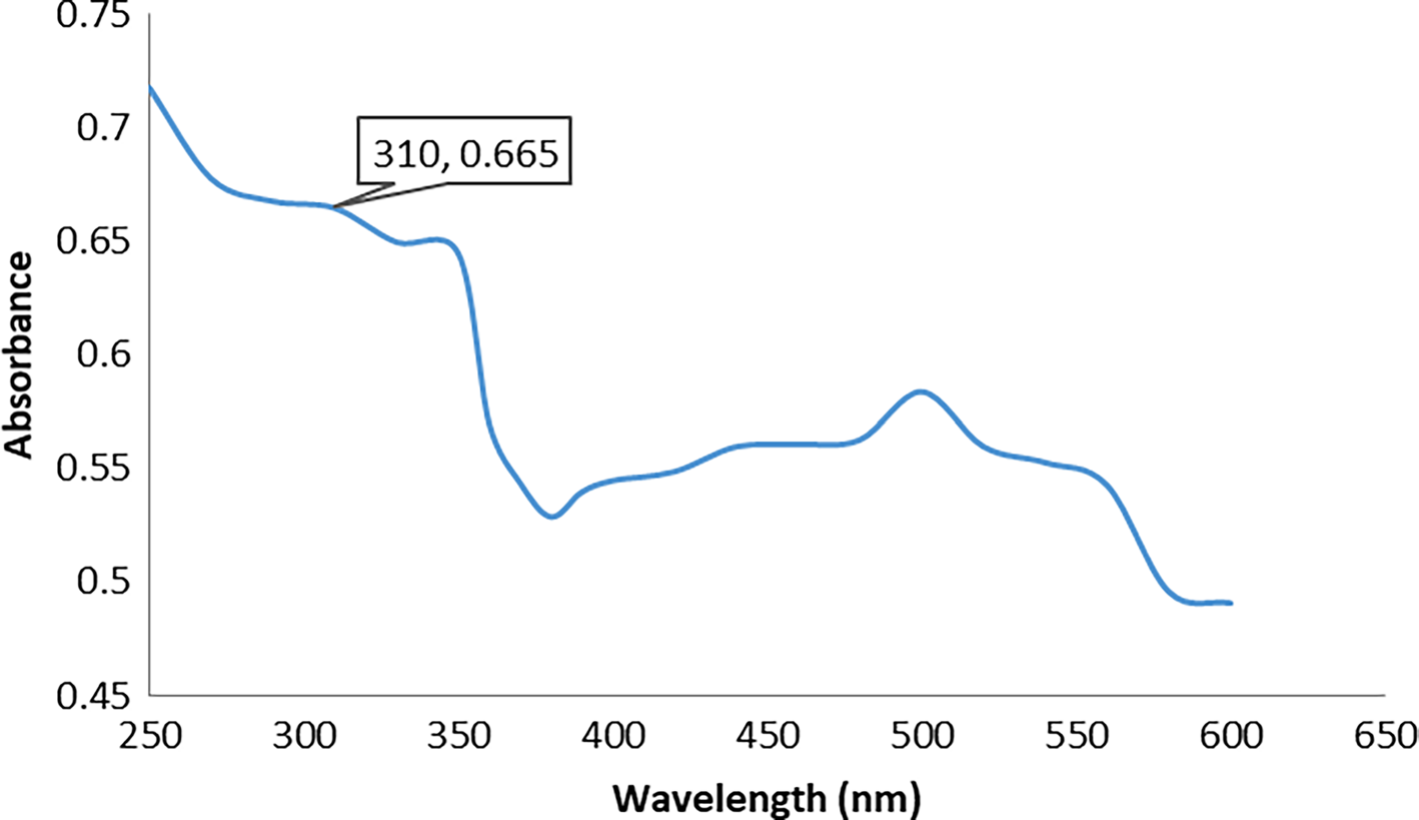
UV–Vis spectroscopy of ZnO nanoparticles. Direct magnification equals 60,000 × and high voltage equals 80 kv

UV–Vis spectroscopy is a susceptible technique that can detect the formation of metallic nanomaterial. The ultraviolet spectra of green-synthesized ZnO NPs indicated that ZnO NPs have characteristic peaks between 300 and 350 nm (Fig. 1). The sample’s distinctive peak can be seen at 310 nm [32].

#### X-Ray Diffractometer (XRD) Analysis

**Fig. 2:**
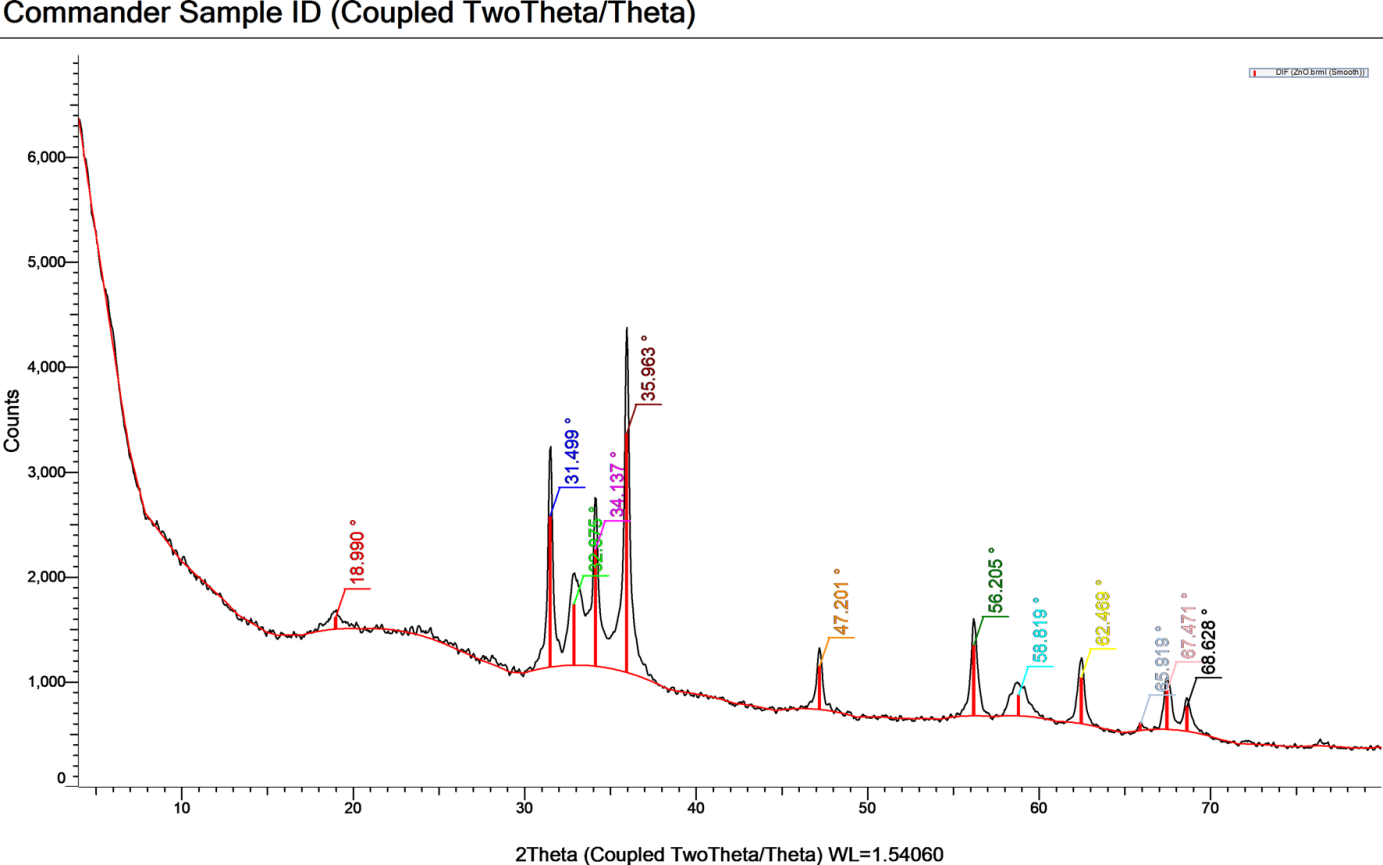
XRD Pattern of ZnO NPs

Figure 2 illustrates the crystalline structure of green-synthesized ZnO NPs which were determined by XRD technique. The peaks at 2 *θ* = of 31.49°, 32.88°, 34.14°, 35.96°, 47.20°, 56.21°, 58.82°, 62.47°, 65.92°, 67.47°, and 68.63° were corresponding to the crystal planes 100, 002, 002, 101, 102, 110, 110, 103, 200, 201, and 202 of ZnO NPs, indicating that they possess a hexagonal zincite-type crystal. A characteristic peak at 18.99° was detected, suggesting the presence of egg albumin. The average crystallite size of ZnO NPs was found to be approximately 18.2 nm. The crystallite/particle size was calculated using the Debye–Scherrer equation for all diffraction peaks.

#### TEM Micrographs of ZnO NPs

**Fig. 3:**
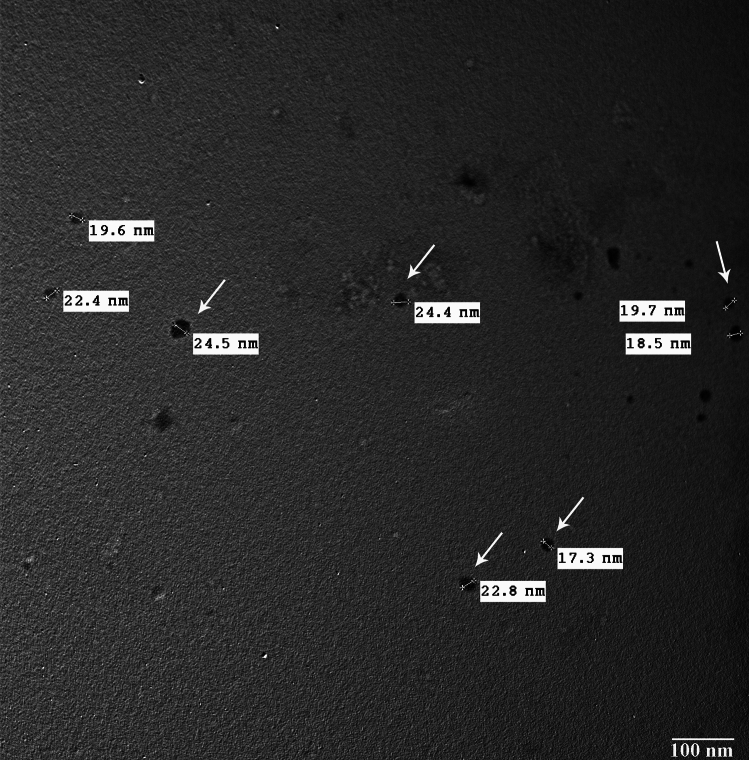
TEM image of ZnO nanoparticles synthesized using egg white

The TEM micrographs showed the internal structure of ZnO nanocrystals as homogeneously aggregated polygonal ZnO nanoparticles with an average size of ~ 20 nm which is in agreement with the XRD result (Fig. 3).

### Toxicity Study

Results indicated that ZnO NPs up to a dosage of 600 mg/kg did not produce any mortality. The median effective dose (ED_50_) was selected based on the proposed LD_50_ obtained from the acute toxicity study. Two doses were selected which are 1/10 and 1/20 of the proposed LD_50_, that is, 600 mg/kg body weight.

### Recovered Larvae from the Liver

#### Larvae Counting

**Fig. 4:**
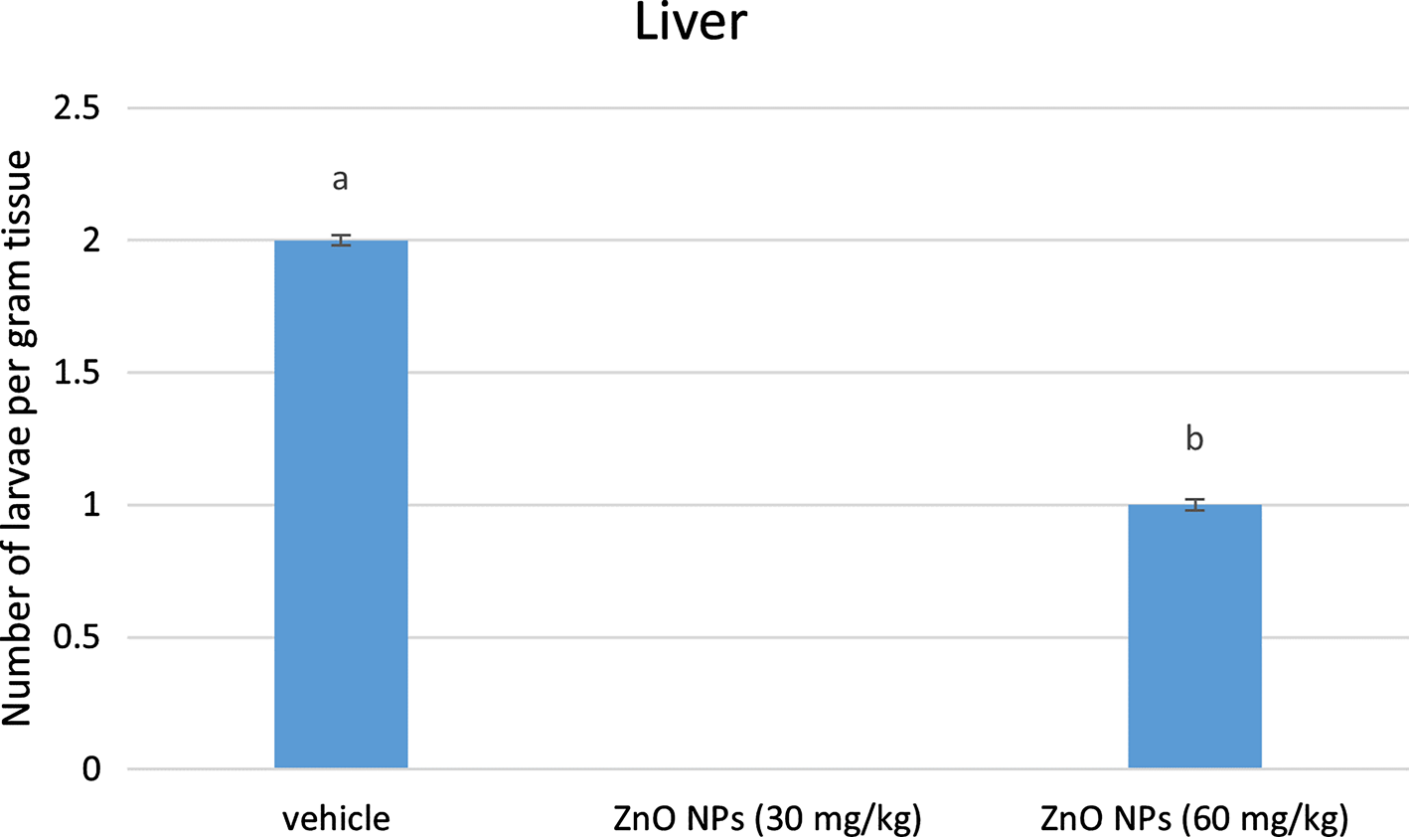
Larval counts in the infected liver

Figure 4 shows the effect of different concentrations of ZnO NPs (30 and 60 mg/kg) on rats infected with *P. equorum* larvae after 10 days of administration. The total number of larvae recovered per gram of tissue was two larvae. Furthermore, no larvae were recovered from (30 mg/kg) the treated group and one larva per g. tissue was recovered from the ZnO NPs (60 mg/kg)-treated group compared to untreated infected animals.

#### Scanning Electron Microscopy (SEM) of *P. equorum* Larvae

**Fig. 5:**
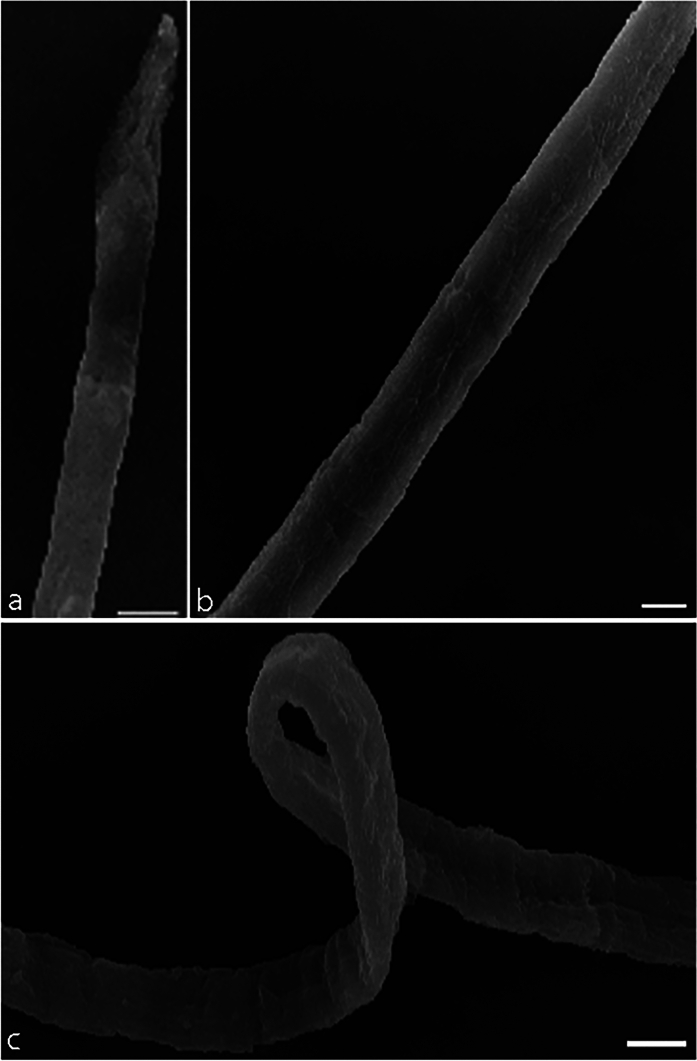
SEM micrograph of extracted larvae from experimentally infected rats with *P. equorum*. Scale bar 10 μm

**Fig. 6:**
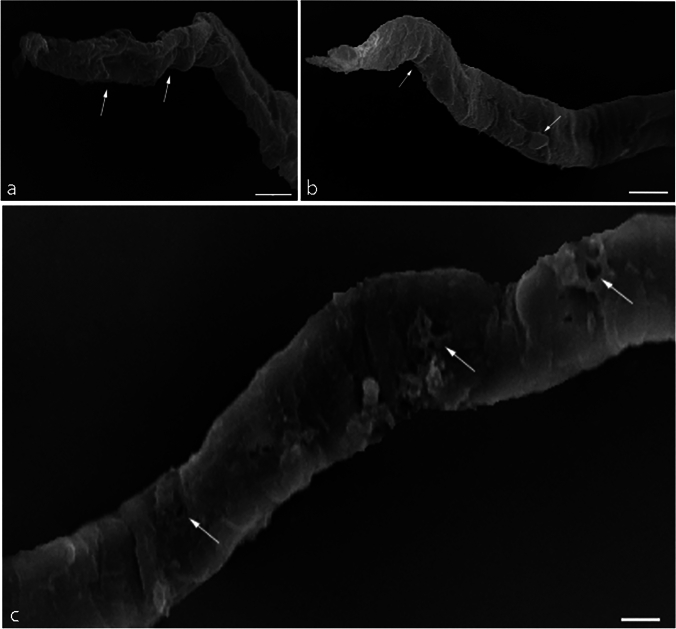
SEM micrograph of extracted larvae from experimentally infected rats with *P. equorum* following treatment with ZnO NPs (30 mg/kg). Scale bar 10 μm

**Fig. 7:**
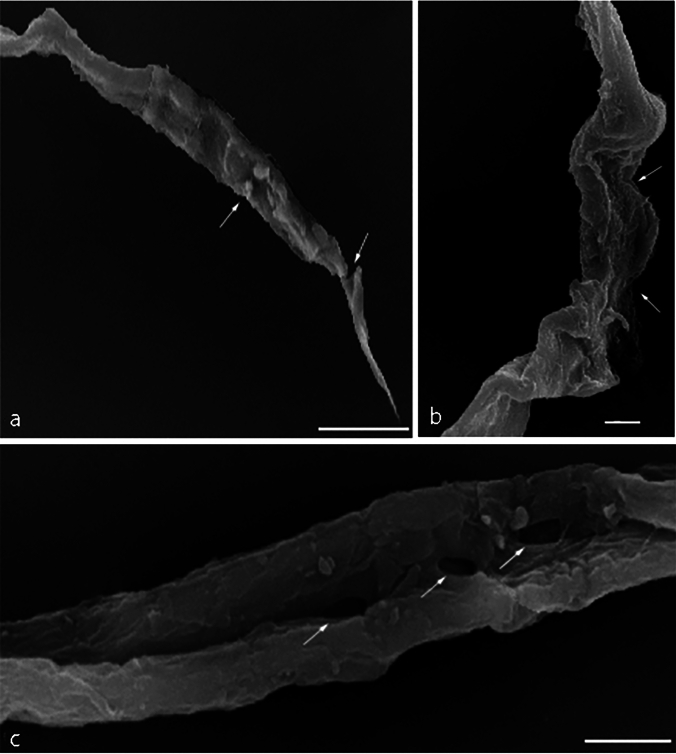
SEM micrograph of extracted larvae from experimentally infected rats with *P. equorum* following treatment with ZnO NPs (30 mg/kg). Scale bar 10 μm

SEM micrographs of *P. equorum* larvae are shown in Figs. 5, 6, and 7. Recovered larvae from untreated infected animals showed normal cuticles and smooth bodies with no morphological or structural alterations as shown in Fig. 5. On the other hand, conspicuous changes were observed in the larval cuticle after treatment with different concentrations of ZnO NPs (30, 60 mg/kg). Recovered larvae from low-dose ZnO NPs (30 mg/kg) animals revealed morphological deformations. The larvae had cuticular damage and areas with multiple blebs, pores, and loss of normal creases. Moreover, the cuticle of the treated larvae turned opaque as shown in Fig. 6. In comparison, recovered larvae from high dose ZnO NPs (60 mg/kg) animals showed severe alterations in the body shape and cuticle surface of the larvae. The larvae had lost their cylindrical form due to dehydration of the body. In addition, it showed severe erosion, shrinkage of the cuticle layers, and sloughing off some areas of the cuticle compared to the untreated larvae as shown in Fig. 7.

### Effect of ZnO NPs on Biochemical Parameters

Data shown in Table 1 represented that *P. equorum* infection caused significant elevation (*p* < 0.05) in AST, ALT, ALP, and GGT activities compared to the control group. Furthermore, ZnO NPs administration showed a non-significant change (*p* > 0.05) in previous enzymes compared to the control group. Regarding the therapeutic effect of ZnO NPs, the activities of AST, ALT, ALP, and GGT revealed a significant decrease (*p* < 0.05) compared to the infected untreated animals. As regards total protein, albumin, and bilirubin concentrations, there was a non-significant difference (*p* > 0.05) in the model group compared to the control animals. In addition, ZnO NPs the administration showed a non-significant change (*p* > 0.05) in total protein, albumin, and bilirubin concentrations compared to the control group. Also, exposure to ZnO NPs treatment did not cause significant change (*p* > 0.05) in the previous parameters compared to the model group. In contrast, globulin concentration showed a significant increase (*p* < 0.05) of the model group compared to the control animals. Besides, ZnO NPs administration showed non-significant change (*p* > 0.05) in globulin concentration compared to the control group. Treatment with ZnO NPs showed a significant decline (*p* < 0.05) in globulin concentration compared to the infected untreated group, as shown in Table 1. Table 1 shows a significant rise (*p* < 0.05) in total cholesterol (TC), triglycerides (TG), and low-density lipoprotein (LDL) levels of the infected untreated animals and a significant decrease (*p* < 0.05) in high-density lipoprotein (HDL) level compared to control group. At the same time, oral administration of ZnO NPs (30 and 60 mg/kg) caused a non-significant change (*p* > 0.05) in TC, TG, LDL, and HDL levels when compared to the control group. Contrarily, treatment with ZnO NPs caused a significant dip (*p* < 0.05) in TC, TG, and LDL levels and a significant elevation (*p* < 0.05) in HDL level compared to the model group. Data recorded in Table 1 demonstrated that a significant drop (*p* < 0.05) were observed in hemoglobin (Hb) concentration and butyrylcholinesterase (BCHE) activity of the model the group compared to control animals. Also, ZnO NPs administration showed a non-significant change (*p* > 0.05) in previous parameters compared to the control group. In contrast, ZnO NPs (30, 60 mg/kg) treatment caused a significant elevation (*p* < 0.05) in Hb concentration and BCHE activity compared to the infected untreated group.

**Table 1:** Therapeutic effect of ZnO NPs on biochemical parameters in *P. equorum*-infected rats

Parameters	Noninfected groups			Infected groups		
	Control	ZnO NPs (30 mg/kg)	ZnO NPs (60 mg/kg)	Vehicle	ZnO NPs (30 mg/kg)	ZnO NPs (60 mg/kg)
AST(U/mL)	140.20 ± 4.66	149.17 ± 3.94	141.00 ± 4.51	191.02^a^ ± 8.40	172.60^b^ ± 8.40	160.33^b^ ± 4.36
ALT(U/mL)	48.60 ± 1.93	48.17 ± 1.85	44.67 ± 3.33	59.60^a^ ± 2.26	53.33^b^ ± 4.78	51.50^b^ ± 3.45
ALP(U/L)	155.00 ± 10.73	162.50 ± 11.25	165.60 ± 10.80	226.20^a^ ± 7.43	187.80^b^ ± 8.91	172.40^b^ ± 9.79
GGT(U/L)	2.71 ± 0.12	3.12 ± 0.18	2.94 ± 0.14	5.10^a^ ± 0.12	3.78^b^ ± 0.16	3.59^b^ ± 0.20
Total protein (g/dL)	5.03 ± 0.05	5.03 ± 0.07	4.95 ± 0.09	4.98 ± 0.06	4.92 ± 0.07	4.99 ± 0.07
Albumin (g/dL)	3.28 ± 0.19	3.33 ± 0.20	3.08 ± 0.03	3.20 ± 0.05	2.97 ± 0.13	3.52 ± 0.16
Globulin (g/dL)	1.54 ± 0.10	1.70 ± 0.15	1.77 ± 0.15	2.03^a^ ± 0.04	1.85^b^ ± 0.13	1.66^b^ ± 0.11
Bilirubin (mg/dL)	0.12 ± 0.01	0.15 ± 0.01	0.14 ± 0.00	0.12 ± 0.01	0.11 ± 0.02	0.13 ± 0.01
Cholesterol (mg/dL)	120.53 ± 2.62	134.58^a^ ± 2.06	129.38^a^ ± 3.35	154.11^a^ ± 2.38	136.43^b^ ± 4.89	135.08^b^ ± 5.19
Triglycerides (mg/dL)	90.10 ± 0.80	78.00^a^ ± 4.41	64.78^a^ ± 1.33	103.37^a^ ± 2.33	89.76^b^ ± 4.08	69.44^b^ ± 2.02
LDL (mg/dL)	36.22 ± 2.05	39.82 ± 1.76	33.43 ± 0.95	48.35^a^ ± 1.98	43.40^b^ ± 0.84	42.07^b^ ± 2.25
HDL (mg/dL)	71.17 ± 2.04	83.39^a^ ± 3.88	81.80^a^ ± 1.19	52.38^a^ ± 2.08	69.89^b^ ± 0.91	82.05^b^ ± 1.59
Hb (g/dL)	8.78 ± 0.09	8.40 ± 0.06	8.68 ± 0.08	4.66^a^ ± 0.15	6.96^b^ ± 0.24	6.62^b^ ± 0.20
BCHE (µmol/min/mL)	248.42 ± 12.00	265.28 ± 15.15	238.57 ± 1.19	226.10^a^ ± 5.33	252.62^b^ ± 13.84	249.46^b^ ± 6.94

Results are expressed as mean ±SE in each group

^a^ significant at (*P* < 0.05) compared to control group

^b^ significant compared to vehicle group

### Effect of ZnO NPs on Oxidative Stress Markers

A significant increase (*p* < 0.05) was observed in the MDA concentration and NO level of the model group compared to the control group. Whereas, oral administration of ZnO NPs (30, 60 mg/kg) showed non-significant change (*p* > 0.05) in MDA concentration and NO level compared to the control animals. While a significant decrease (*p* < 0.05) was determined in the MDA concentration and NO level after treatment with ZnO NPs (30, 60 mg/kg body weight, orally) compared to the infected untreated the group as shown in Table 2. Data recorded in Table 2 represent a significant decrease (*p* < 0.05) in GSH, CAT, and GST levels in the model group compared to the control group. Regarding the effect of ZnO NPs (30, 60 mg/kg), GSH, CAT, and GST levels revealed a non-significant change (*p* > 0.05) compared to the control group. In contrast, rats treated with ZnO NPs (30, 60 mg/kg) showed a significant elevation (*p* < 0.05) in GSH, CAT, and GST levels compared with the infected untreated group.

**Table 2:** Therapeutic effect of ZnO NPs on oxidative stress markers in *P. equorum*-infected rats

Parameters	Noninfected groups			Infected groups		
	Control	ZnO NPs (30 mg/kg)	ZnO NPs (60 mg/kg)	Vehicle	ZnO NPs (30 mg/kg)	ZnO NPs (60 mg/kg)
MDA (nM/g.tissue)	2.75 ± 0.12	2.94 ± 0.04	2.85 ± 0.15	3.96^a^ ± 0.14	3.61^b^ ± 0.12	2.86^b^ ± 0.03
GSH (mM/g.tissue)	4.71 ± 0.05	4.86 ± 0.17	4.62 ± 0.10	2.31^a^ ± 0.03	3.62^b^ ± 0.14	4.11^b^ ± 0.16
CAT (U/min/g.tissue)	164.66 ± 4.70	159.88 ± 3.26	151.82 ± 2.59	105.68^a^ ± 1.93	130.07^b^ ± 4.12	140.31^b^ ± 5.87
NO (μM/g.tissue)	554.63 ± 36.26	625.19 ± 29.28	684.22 ± 39.41	1,290.34^a^ ± 41.51	1,001.03^b^ ± 113.80	895.93^b^ ± 77.84
GST (μM/g.tissue/min)	5.35 ± 0.08	4.87 ± 0.09	5.09 ± 0.07	2.22^a^ ± 0.06	3.48^b^ ± 0.10	4.51^b^ ± 0.07

Results are expressed as mean ±SE in each group

^a^ significant at (*P* < 0.05) compared to control group

^b^ significant compared to vehicle group

### Histopathology

**Fig. 8:**
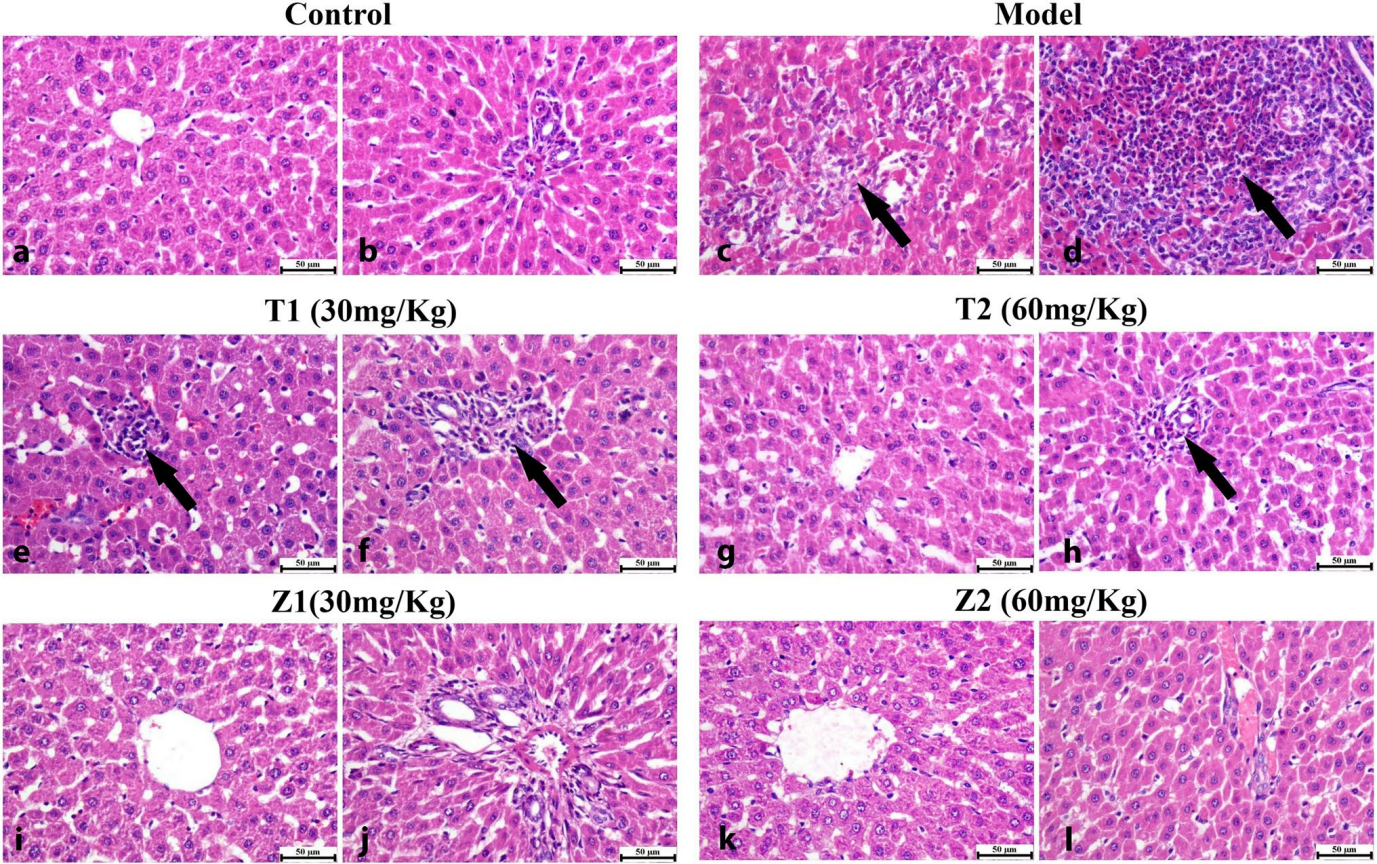
Photomicrographs of the liver (H&E) showing control group exhibiting **a** normal centrilobular area and **b** normal periportal hepatocytes. Model group showing **c** hepatocellular necrosis with mononuclear inflammatory cells infiltration (arrow), **d** intense mononuclear and eosinophilic cells infiltration (arrow). T1 (30 mg/kg) group **e** focal mononuclear inflammatory cells infiltration (arrow), **f** portal infiltration with mononuclear inflammatory cells (arrow). T2 (60 mg/kg) group **g** normal centrilobular hepatocytes, **h** mild portal infiltration with mononuclear inflammatory cells. Z1 (30 mg/kg) group showing normal **i** centrilobular area and **j** normal periportal area. Z2 (60 mg/kg) shows normal **k** centrilobular hepatocytes and **l** periportal area

**Fig. 9:**
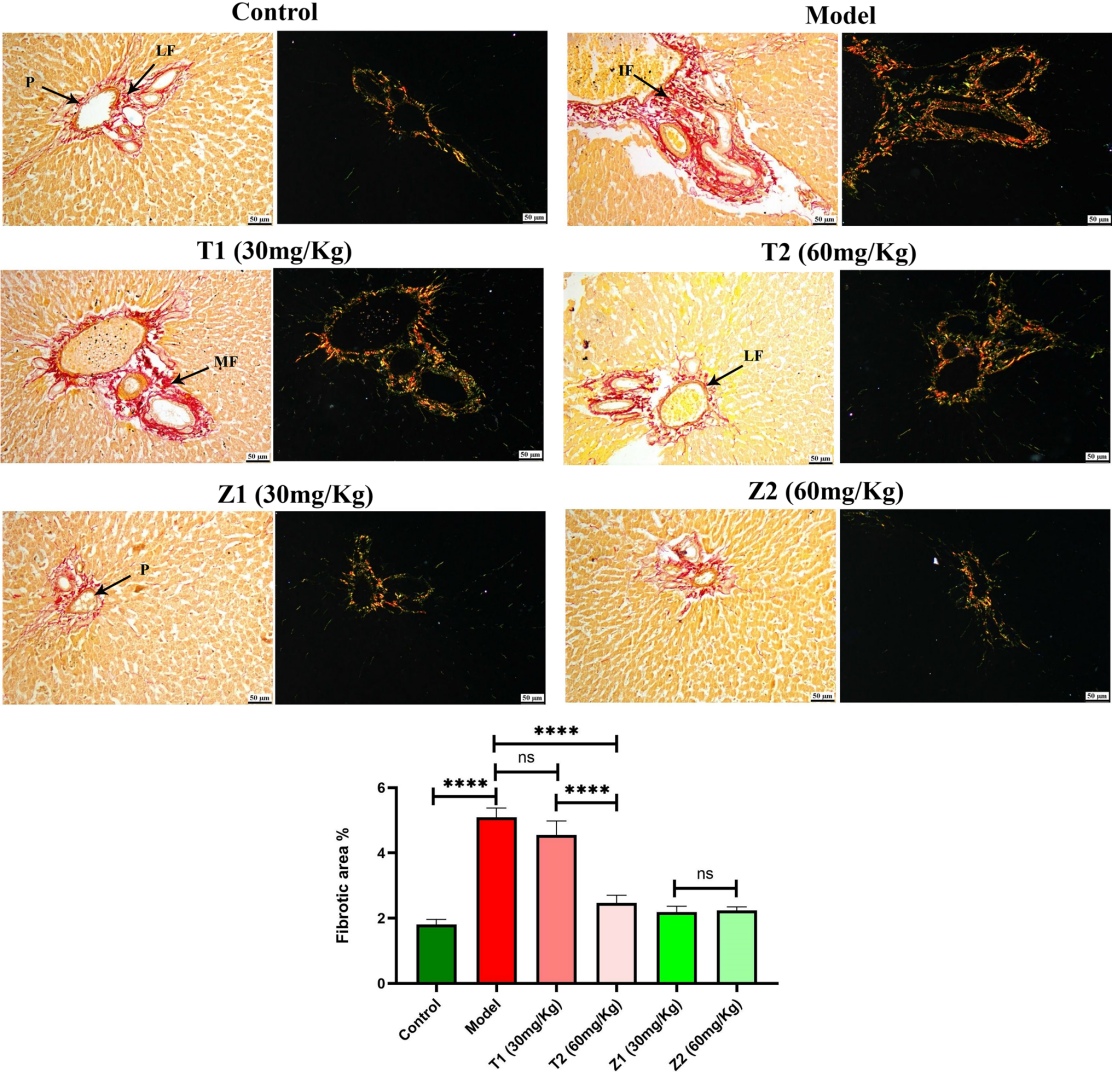
Photomicrographs of the liver (PSR in bright field and polarized light). Control group showed a normal limited amounts of fibroblasts (LF) in the portal area (*P*). Model group exhibiting increased amounts of fibroblasts (IF). T1 group showing moderate amounts of fibroblasts (MF). T2, Z1, and Z2 exhibited a normal limited amount of fibroblasts (LF). The chart illustrates fibrotic area as (area %), data were presented as means ± SE, and significance was considered at *p* < 0.05

As illustrated in Fig. 8, microscopic examination of liver sections from the control group revealed the normal histologic structure of hepatic parenchyma. Hepatocytes were normal in both the centrilobular and periportal areas. The portal areas were normal as well. The vehicle group showed wide multifocal areas of hepatocellular necrosis that exhibited intense mononuclear and eosinophils infiltrations. Portal areas showed severe mononuclear inflammatory cells with widespread necrosis in periportal hepatocytes. Moderate improvement was noticed after treatment with ZnO NPs (30 mg/kg), and small sporadic focal areas of necrotic hepatocytes were occasionally seen in the hepatic parenchyma with mononuclear inflammatory cell aggregations. The portal areas exhibited mild mononuclear inflammatory cell infiltration. ZnO NPs (60 mg/kg) the group showed marked improvement; both centrilobular and periportal areas were normal in most of the examined sections, except for fewer portal mononuclear inflammatory cell infiltration. While, oral administration of ZnO NPs (30, 60 mg/kg) showed normal liver tissue without any detectable alterations. Picrosirius red (PSR)-stained sections (Fig. 9) in both bright field and polarized light revealed normal limited amounts of fibroblasts (LF) in the portal areas (*P*) of control group. Meanwhile, the vehicle group showed increased amounts of fibroblasts (IF) in the portal tirade. Treatment with ZnO NPs (30 mg/kg) showed moderate amounts of fibroblasts (MF) in the portal area, while ZnO NPs (60 mg/kg) showed a normal limited amounts of fibroblasts (LF). Though oral administration of ZnO NPs (30, 60 mg/kg) exhibited apparently normal portal areas without fibroplasia. A significantly higher value of the fibrotic area was detected in vehicle group in comparison with other experimental groups. No significant difference was observed between ZnO NPs (30 mg/kg)-treated and vehicle group; meanwhile, a significant reduction in fibrosis was detected in ZnO NPs (60 mg/kg)-treated group.

### Immunohistochemistry

#### Expression of NF-κβ

**Fig. 10:**
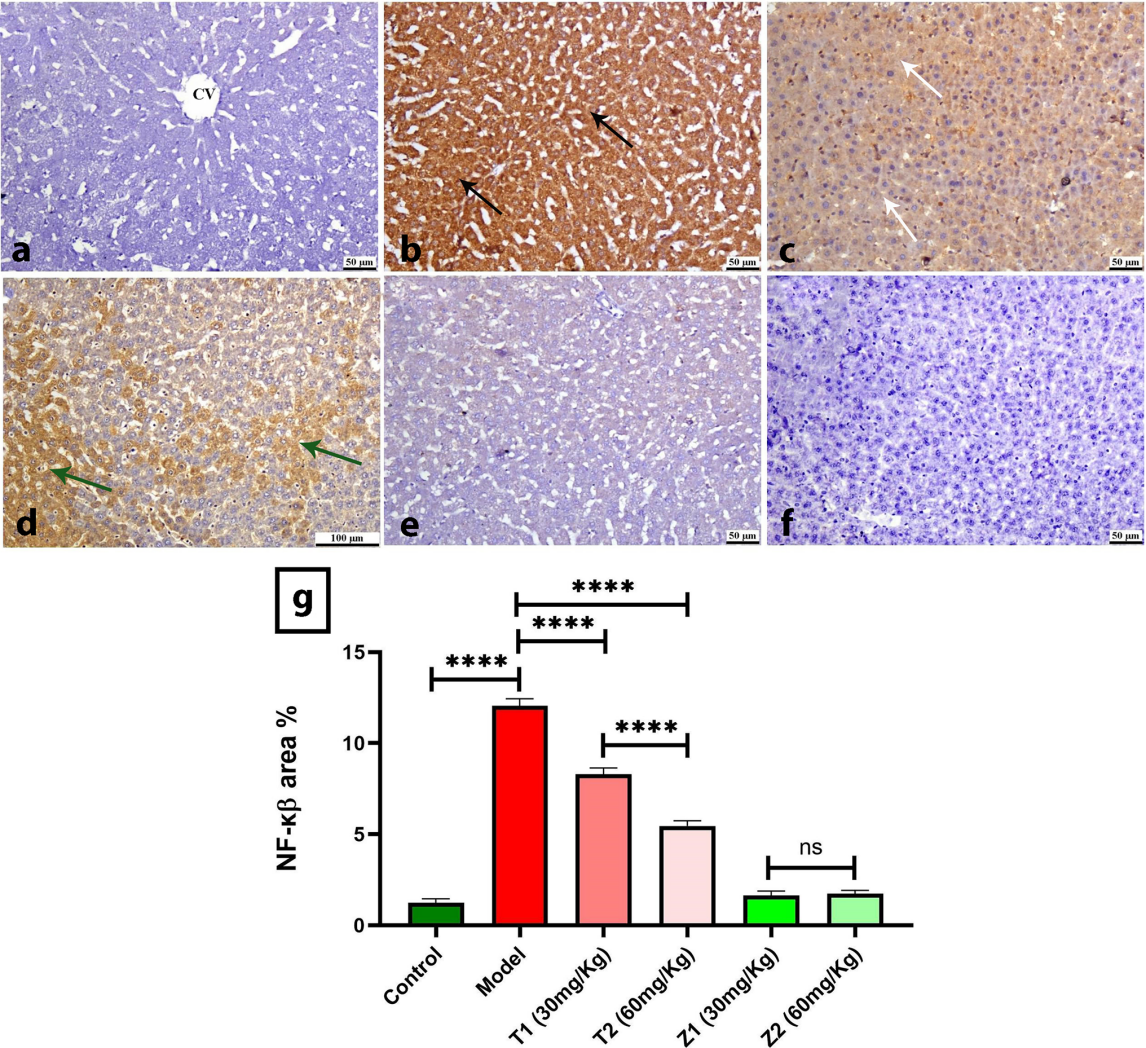
Photomicrographs of liver (Immune staining) showing **a** control group, normal negative to limited NF-κβ staining, **b** Model group, intense hepatic NF-κβ staining (black arrow), **c** group, moderate positive expression (white arrow), **d** T2 group, mild positive staining (green arrow), **e** Z1 and **f** Z2 groups exhibiting normal amounts of NF-κβ. **g** Charts represent quantified NF-κβ expression as area %. Data were presented as means ± SE. Significance was considered at *p* < 0.05 (colour figure online)

The control group showed normal negative to limited hepatic expression of NF-κβ; meanwhile, the model group exhibited a marked increase in its positive staining. Treatment with ZnO NPs (30, 60 mg/kg) showed a reduction in NF-κβ with increasing the dose. While oral administration of ZnO NPs (30, 60 mg/kg) exhibited normal levels of hepatic NF-κβ. Quantification of NF-κβ is illustrated, in comparison to the control group, a significantly higher value of NF-κβ was detected in the model group. Treatment with ZnO NPs (30, 60 mg/kg) showed significant reduction in NF-κβ in dose-dependent manner No statistically significant difference was noticed between ZnO NPs (30, 60 mg/kg)-administrated groups and the control group (Fig. 10).

## Discussion

Equine ascariasis is a global endoparasitic disease caused by *Parascaris* spp. that predominantly affects the health status of grazing horses and donkeys [33]. Infected animals with *P. equorum* may develop some respiratory symptoms, ill thrift accompanied by rough hair coat and bouts of diarrhea or colic [34]. Moreover, severe infestations of adult *P. equorum* can cause small intestine rupture and death [35]. Traditional control strategies mainly rely on three major drug classes: benzimidazoles (BZ), the tetrahydropyrimidine pyrantel (PYR), and macrocyclic lactones (ML) [36]. Although the anthelmintic agents are very effective in most situations, the emergence of drug resistance has been recorded in many cases, especially in *P. equorum* infection. Consequently, the demand for alternative medical agents with different mechanisms is an urgent necessity, which can be achieved by nanoparticle-based drug formulations. Old-style chemical methods used for nanomaterial synthesis leads to the adsorption of toxic compounds onto the surface of synthesized NPs. Therefore, this study sheds light on the therapeutic effect of green-synthesized ZnO NPs on *P. equorum*-infected rats. Parasitic infection in the hepatic cells causes significant morbidity and mortality, leading to recurrent cholangitis, cirrhosis, liver failure, and cancer [37]. Determination of enzyme levels such as AST, ALT, ALP, and GGT is mainly used as biomarkers for liver damage. The current investigation revealed that inoculation of *P. equorum* eggs showed a marked elevation in AST, ALT, ALP, and GGT activities. Larval infestation in the liver resulted in destruction and impairing cell membrane permeability in the hepatocytes. Subsequently, destructed hepatocytes liberate excessively the hepatic enzymes into the blood stream and elevating their levels in the serum [5]. The decline of serum transaminases to near normal levels by the treatment with ZnO, NPS indicates a possible regeneration of hepatocytes and healing effect on the hepatic parenchyma [38]. Globulins are a group of proteins that help transport nutrients and fight infections. Globulins consist of three fractions: α and β globulins are synthesized by the liver, whereas gamma globulins (immunoglobulins) are antibodies produced by lymphocytes and plasma cells in lymphoid tissue [39]. Gamma globulins can recognize a broad spectrum of specific antigenic determinants, constituting the basis of the humoral immune response [40]. In the current study, there was an increase in globulin concentration in *P. equorum*-infected rats. In agreement with our results, Bordoloi *et al*. [41] observed the members of globulin are mainly positive acute phase proteins; therefore, their concentration increases during inflammation and parasitic infection in livestock. On the other hand, the decrease in globulin concentration near to the normal level following NPS administration may be due to the absence of infective agent (*P. equorum* larvae) that irritates the immune cells of lymphoid organs [42, 43]. Anemia is abnormal hemoglobin (Hb) concentration in blood in which the oxygen-carrying capacity of the red blood cells is reduced and is insufficient to meet the various physiologic needs. There are various causes of anemia among which iron deficiency is the most common cause followed by folate, vitamin B12, vitamin A deficiencies, and parasitic infections [44]. Intestinal parasitic infection causes anemia by reducing iron uptake from the intestine, directly sucking blood, and interfering directly and indirectly in iron metabolism [45]. Al-Daraji and Al-Amery [46] reported that the significant decline in the concentration of Hb may correlate with the activity of *P. equorum* larvae in the penetration of small intestine mucosa and rupture of blood vessels. Additionally, the fall in Hb concentration might be due to metabolic disturbance caused by worms rather than a direct blood loss [47]. However, the elevation of Hb concentration to near normal levels by the treatment with ZnO NPs in the current investigation indicates the anthelmintic effect of nanomaterials. Free radicals’ production from the zinc surface is leading to an increase in oxidative stress and membrane damage of *P. equorum* larvae, causing a drop in the activity and mobility of larvae [38]. Butyrylcholinesterase (BChE) is a bio-scavenging enzyme in serum, synthesized by the liver, and exhibits antioxidant and anti-inflammatory activities. It hydrolyzes ester-containing compounds and acts as a scavenger against neurotoxic organophosphates (OPs) [48]. BChE concentration showed a significant decrease in all infected animals that are in agreement with Farid and Horiia [49]recorded that nematodes infection makes rats more susceptible to OP toxicity through increasing various pro-inflammatory cytokines (IL-1, IL-6, and TNF-). A remarkable increment in BChE activity in NPs-treated animals were recorded, which is in agreement with Pang *et al*. [50], who indicated that nanomaterials could act as antidotes against OP poisoning. An elevated level of cholesterol, triglycerides, and LDL and the decrement in HDL level in the current study might be due to parasitic stress in infected rats, which caused an increase in the output of epinephrine and corticosteroids [5]. In the same line, Szewczyk-Golec *et al*. [51] indicated that the toxicity effects of gastrointestinal parasites caused an enlargement in liver and intestines hence leading to elevate lipid levels. Otherwise, the reduction in cholesterol, triglycerides, and LDL levels and increasing HDL level following NPS administration may be due to a decline in numbers of larvae consequence of decreasing parasitic strain in the host. It is also congruent with the findings of Mobarez *et al*. [12], who revealed that zinc is considered an integral part of some metalloenzymes that are severed in lipid digestion and absorption. Zinc supplementation also increases hepatocyte activity and improves lipid metabolism in the liver [52]. Reactive oxygen species (ROS) or reactive nitrogen species (RNS) are produced as normal products during various processes in the cells and tissues of the body. ROS/RNS immoderate formation over than antioxidant defenses can create oxidative stress status. Recent studies have suggested intestinal parasitic infection as toxocariasis and ascariasis can stimulate excessively production of free radicals such as superoxide and other intermediate products of free radical activity such as hydrogen peroxide in mammalian tissues, accordingly, provoke the occurrence of oxidative stress [53]. Lipid peroxidation (LP) is a complex chain reaction that is created by free oxygen radicals influencing unsaturated fatty acids in cell membranes, leading to their damage. Therefore, LP is the best indicator of ROS levels that induced systemic biological damage [54]. Estimation of malondialdehyde (MDA) level evaluates the degree of LP and the level of ROS indirectly as MDA is the end product of lipid peroxidation [55]. Nitric oxide (NO) is a biological mediator with diverse roles in host defense and homeostasis when generated at a low level for a brief period but becomes genotoxic mutagenic when generated at higher concentrations for a long time [56]. The present study revealed that *P. equorum* inoculation significantly caused an elevation in the MDA and NO levels. Presence of parasitic infections destroyed the tissue, lipid peroxidation took place in a large volume, MDA as the most important product of this process elevated and fascinated demolition of cell structures, inflammation and necrosis [54]. In the same line, Nazarlu *et al*. [45] reported that invasion of *Toxoplasma* parasite induces NO production by macrophages to maintain the viability of the cell during its residence. In addition, *F. hepatica* infection is accompanied by stimulation of leukocytes and generation of the superoxide radical and NO [57]. However, treatment with ZnO NPs in the present study significantly decreased the MDA and NO levels compared to the infected group, suggesting the ability of ZnO nanomaterial to scavenge free radicals and mediate the oxidative stress state [58]. Furthermore, Alajmi *et al*. [13] concluded that green-synthesized nanoparticles served as highly potent and novel therapeutic agents for the quenching of NO. Also, it regulated the pathological conditions caused by the unwarranted generation of NO and its oxidation product—peroxynitrite. The antioxidant mechanism has evolved to protect biological systems against oxidative stress through quenching free radical reactions and inhibiting cellular damage, which includes enzymatic scavengers such as glutathione-s-transferase (GST) and catalase (CAT) in addition some small antioxidant molecules such as GSH. Stimulation of antioxidant enzymes such as dismutase, peroxidase, CAT, and GSH was observed during experimental trichinellosis in mice [57]. The present study has shown that *P. equorum* infection induced a significant decrease in GSH, CAT, and GST levels that is in agreement with Kolodziejczyk *et al*. [59] who referred that the GSH level is decreased during fasciolosis infection that may be due to the enhanced oxidation of GSH into glutathione disulfide catalyzed by free radicals. A decrease in GSH level was also detected in patients with toxoplasmosis, which suggests the occurrence of changes in the oxidant–antioxidant balance as a mechanism of tissue damage [51]. Furthermore, GSH depletion was attributed to the increased cytotoxicity with H_2_O_2_ produced due to inhibition of glutathione reductase that keeps glutathione in the reduced state [60]. Also, Dkhil *et al*. [61] reported that helminth infection serves to decrease the hepatic antioxidant capacity and generate lipid peroxides. Contrarily, ZnO NPs administration to the infected rats raised GSH, CAT, and GST levels that are in accordance with Bauomy [62], who concluded that ZnO NPs possess excellent anti-parasitic activity. In the same manner, Dkhil, *et al*. [63] reported that the nanoparticles of ZnO revealed anticoccidial and antioxidant activities. Host adaptation is highly evolved among parasitic worms and there is ample evidence that helminths manipulate the immune response in ways that prolong their survival in the host or promote their dispersal in the susceptible host population. Eosinophilia is an increase in the number of eosinophils in the blood or tissues, that participate in the defense against helminth parasites and in hypersensitivity reactions in mammals [64]. In this study, we investigate the role of eosinophils during *P. equorum* infection and show that they are beneficial to the host by promoting an effective immune response that partially controls liver damage. Accumulation of eosinophils is a well-documented feature of helminth infections since these cells can be vital to develop Th2 immune responses against parasitic helminths [65]. Host nuclear factor-kappa B (NF-κB) transcription factor plays a pivotal role in innate immunity and resistance to infection. It induces the expression of several genes that encode pro-inflammatory cytokines. It also participates in regulating the differentiation and survival of innate immune cells and lymphocytes. Infection of host cells with pathogens usually activates host NF-κB signaling pathways. Most parasites evolved diverse protective mechanisms against NF-κB activity to shield their continued existence [66].

## Data Availability

All data generated or analyzed during this study are included in this published article.
